# Proteostatic defect drives biophysical remodeling that triggers cell competition

**DOI:** 10.1016/j.isci.2026.116804

**Published:** 2026-07-14

**Authors:** Wonjae Song, Mai Yukitake, Kei Kozawa, Nanami Sato, Jiaying Wen, Zehao Dai, Susumu Ishikawa, Akifumi Shiomi, Hirofumi Shintaku, Seiichiro Ishihara, Hisashi Haga, Yasuyuki Fujita

**Affiliations:** 1 Department of Molecular Oncology, Graduate School of Medicine, Kyoto University, Kyoto 606-8501, Japan; 2 Division of Molecular Oncology, Institute for Genetic Medicine, Hokkaido University Graduate School of Chemical Sciences and Engineering, Sapporo 060-0815, Japan; 3 Institute for Life and Medical Sciences, Kyoto University, Kyoto 606-8507, Japan; 4 The Hakubi Center for Advanced Research, Kyoto University, Kyoto 606-8501, Japan; 5 Department of Advanced Transdisciplinary Sciences, Faculty of Advanced Life Science, Hokkaido University, Sapporo 060-0810, Japan

**Keywords:** cell competition, mammalian cell culture, Ribosomal Protein Rpl24, apoptosis, p53, proteostasis, cellular biophysical alterations

## Abstract

Cell competition is a fundamental tissue-surveillance process in which less-fit “loser” cells are actively eliminated by “winner” neighbors. Here, we establish a mammalian epithelial model of ribosomal protein insufficiency using Madin-Darby canine kidney (MDCK) cells with tetracycline-inducible shRNA targeting ribosomal protein large subunit 24 (Rpl24). When Rpl24-knockdown cells are co-cultured with normal cells, Rpl24-knockdown cells undergo apoptosis through cell competition with surrounding normal cells. Rpl24 knockdown disrupts protein homeostasis, leading to cytoplasmixc protein aggregates. The chemical chaperone 4-phenylbutyric acid (4-PBA) diminishes aggregate accumulation and markedly reduces competitive cell death. Proteostasis disruption also remodels cellular biophysics; Rpl24-knockdown cells exhibit lower homeostatic density, increased cell area, and reduced cell-surface tension. Importantly, these biophysical alterations are reversed by 4-PBA. Together, our findings reveal that ribosomal protein insufficiency links proteostatic stress to biophysical “loser” traits, establishing proteostasis-dependent biophysical remodeling as a key determinant of competitive cell elimination.

## Highlights

• Rpl24-knockdown cells undergo apoptosis via cell competition with normal cells
• Rpl24 knockdown disrupts proteostasis, leading to cytoplasmic protein aggregates
• Proteostasis disruption remodels cellular biophysics such as cell-surface tension
• Proteostasis-dependent biophysical remodeling plays a key role in cell competition

## Graphical abstract

**Figure:**
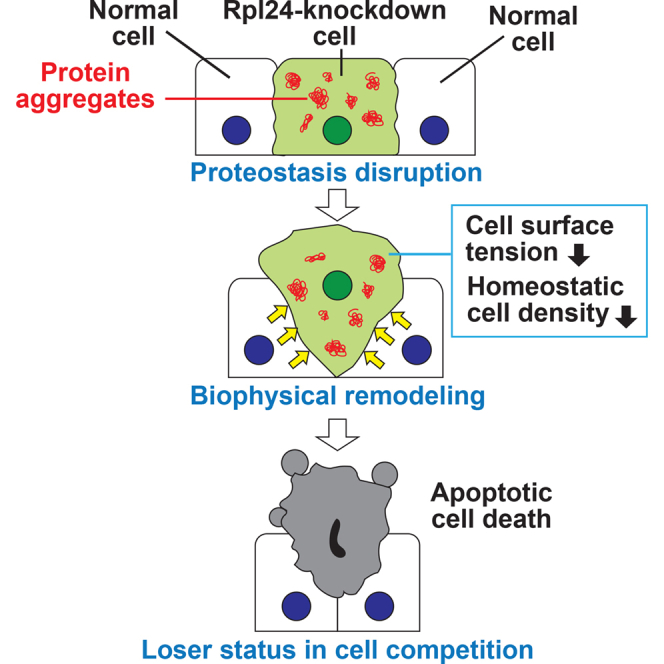

## Teaser

Molecular biology; Cell biology; Organizational aspects of cell biology; Biophysics

## Introduction

A cellular society within epithelial tissues is often exposed to intrinsic and extrinsic insults, and functionally abnormal or aberrant cells continuously emerge during embryonic development and the adult lifetime. Epithelial cells are equipped with several machineries to cope with those potentially harmful cells, and cell competition is one of the most crucial homeostatic processes.^1^^,^^2^^,^^3^^,^^4^^,^^5^^,^^6^^,^^7^^,^^8^ Cell competition is a phenomenon by which cells with different properties compete with each other for survival and space. When abnormal or aberrant epithelial cells appear, they are eliminated from the epithelial layer via cell competition with surrounding normal epithelial cells; abnormal/aberrant cells become losers, while normal cells become winners. In other words, normal epithelial cells have the ability to recognize and actively eradicate those troublesome neighbors.

Cell competition was first discovered in the *Drosophila* wing imaginal disc epithelium.^9^^,^^10^ *Minute* genes encode ribosomal proteins, and *Minute* heterozygous mutant (*M*/+) cells exhibit decreased protein synthesis and cell proliferation. When *M*/+ cells are surrounded by wild-type cells in the wing imaginal disc, *M*/+ cells undergo apoptosis and are eliminated from the epithelial layer. In contrast, when *M*/+ cells alone are present, cell death does not occur, and *M*/+ embryos develop into adult flies. This indicates that the interaction with wild-type cells gives certain cellular stresses to *Minute*-mutant cells, thereby inducing the loser phenotype in cell competition. In addition, recent studies in *Drosophila* have demonstrated that protein homeostasis (proteostasis) becomes disrupted in *Minute*-mutant cells and that the presence of unfolded proteins induces the loser status.^11^^,^^12^ However, the molecular mechanism of how a proteostasis defect triggers cell competition remains enigmatic.

In mammals, ribosomes consist of about 80 ribosomal proteins that form and mature the ribosomes. The genes encoding those ribosomal proteins are extensively distributed over most of the chromosomes,^13^ thus loss of heterozygosity of a given ribosomal gene can readily occur upon chromosomal alterations such as aneuploidy. A previous study suggested that wild-type cells can outcompete ribosomal protein large subunit 24 (Rpl24)-mutated cells in mouse liver during embryonic development,^14^ although the underlying mechanism has remained unexplored.

In this study, using a mammalian cell culture system, we demonstrate that when Rpl24-knockdown cells are co-cultured with normal cells, Rpl24-knockdown cells undergo apoptosis via cell competition with surrounding normal cells. Importantly, proteostasis becomes defective in Rpl24-knockdown cells, which profoundly affects their biophysical properties, thereby triggering cell competition.

## Results

### Knockdown of Rpl24 induces the loser phenotype of cell competition in mammalian epithelial cells

**Figure 1:**
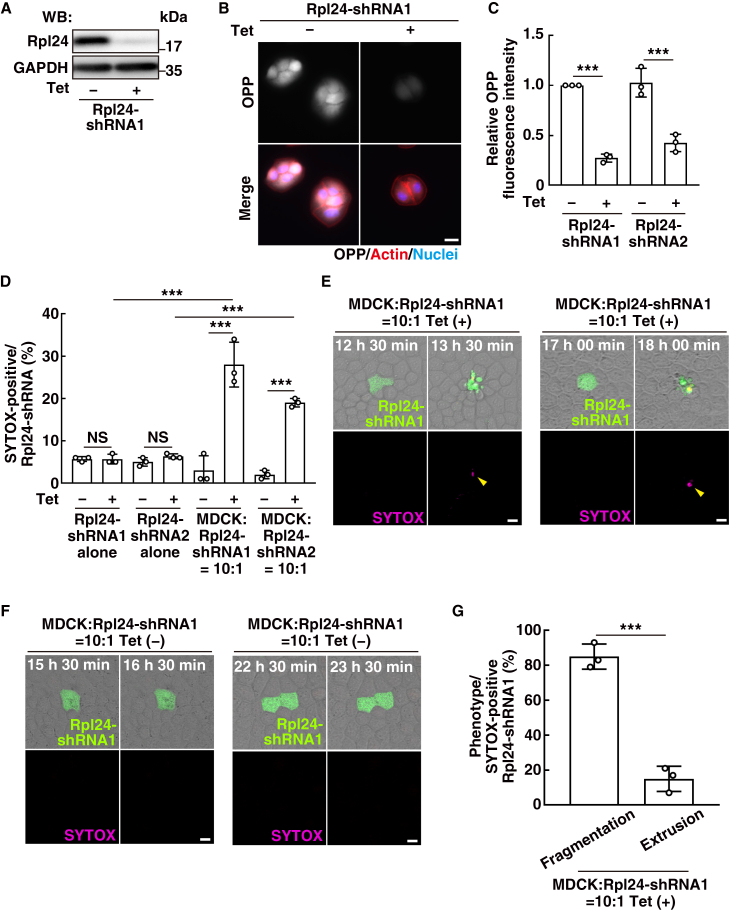
Cell competition between normal and Rpl24-knockdown cells (A) Rpl24 knockdown induced by tetracycline addition in MDCK-pTR Rpl24-shRNA1 cells. MDCK-pTR Rpl24-shRNA1 cells were incubated with or without tetracycline for 72 h, and cell lysates were examined by western blotting using anti-Rpl24 and anti-GAPDH antibodies. (B and C) Effect of Rpl24 knockdown on OPP fluorescence. Rpl24-shRNA1 or -shRNA2 cells were cultured with or without tetracycline at low density for 72 h and stained with OPP (white), Alexa Fluor 568-phalloidin (red), and Hoechst (blue). (C) Quantification of OPP fluorescence intensity. Values are expressed as a ratio relative to Rpl24-shRNA1 (Tet -). Data are mean ± SD from three independent experiments (*n* = 30 cells for each experiment). ∗∗∗*p* < 0.001 (one-way ANOVA with Tukey’s test). (D–F) Cell death of Rpl24-knockdown cells co-cultured with normal cells. (D) Quantification of SYTOX-positive percentage in Rpl24-shRNA cells. Data are mean ± SD from three independent experiments (*n* = 100 cells for each experiment). ∗∗∗*p* < 0.001, NS, not significant (one-way ANOVA with Tukey’s test). MDCK-pTR Rpl24-shRNA1 cells (GFP-positive) were cultured alone or co-cultured with normal MDCK cells in the presence (E) or absence (F) of tetracycline under confluent conditions. Cell death was analyzed with SYTOX dye, and yellow arrowheads indicate dead cells. (G) Quantification of the phenotype of SYTOX-positive Rpl24-knockdown cells. Data are mean ± SD from three independent experiments (*n* = 100 cells for each experiment). ∗∗∗*p* < 0.001 (unpaired two-tailed Student’s *t* test). (B, E, and F) Scale bars, 10 μm. See also Figures S1 and S2.

To elucidate whether and how loss of function of ribosomal proteins induces cell competition in mammals, we established Madin-Darby canine kidney (MDCK) cells stably expressing Rpl24-short hairpin (sh)RNA in a tetracycline-dependent manner. We confirmed by western blotting (Figures 1A, S1A, and S1B) and immunofluorescence (Figures S1C and S1D) that after 72 h of tetracycline addition, the expression level of Rpl24 was reduced by around 50%. To examine the effect of Rpl24 knockdown on protein synthesis, we utilized the O-propargyl-puromycin (OPP) assay to monitor synthesized nascent proteins.^15^ Rpl24 knockdown substantially reduced the OPP fluorescence intensity (Figures 1B and 1C), indicating that Rpl24 knockdown impairs protein synthesis. In addition, the quantification of EdU- or pH3-positive cells showed that Rpl24 knockdown suppressed cell proliferation (Figures S1E–S1H).

We then investigated whether Rpl24 knockdown induces cell competition. After pretreatment with tetracycline for 48 h, MDCK-pTR Rpl24-shRNA cells were co-cultured with normal MDCK cells at a ratio of 1:10, and after 24 h, time-lapse microscopic analysis was conducted. To monitor cell death, we used the SYTOX fluorescent dye, which passes through only damaged cell membranes, binds DNA, and emits fluorescence. We found that around 30% of Rpl24-shRNA cells became SYTOX-positive and were apically extruded during the 24 h time-lapse analysis (Figure 1D). Most of the SYTOX-positive Rpl24-knockdown cells were fragmented just prior to apical extrusion and simultaneously took up SYTOX (Figures 1E and 1G, and Videos S1 and S2), whereas a much smaller fraction of the SYTOX-positive Rpl24-knockdown cells were apically extruded without cell fragmentation (Figure 1G). This observation may indicate that Rpl24-knockdown cells can enter apical extrusion at different stages of cell death progression. When Rpl24 knockdown was not induced in the absence of tetracycline, the cell death of Rpl24-shRNA cells was not frequently observed (Figures 1D and 1F, and Video S3). Furthermore, when Rpl24-knockdown cells were cultured alone, much fewer SYTOX-positive cells were detected (Figure 1D), indicating that the cell death of Rpl24-knockdown cells was induced by cell competition with surrounding normal cells. When Rpl24-knockdown cells were co-cultured with normal cells in a sparse condition, cell death did not frequently occur (Figure S1I). In addition, when a large colony of Rpl24-knockdown cells was surrounded by normal cells, cell death was predominantly observed in Rpl24-knockdown cells directly contacting normal cells, but not in Rpl24-knockdown cells positioned in the center of the colony and not contacting normal cells (Figure S1J). Furthermore, when Rpl24-KD cells were cultured in the conditioned medium collected from co-cultures of normal and Rpl24-KD cells, cell death of Rpl24-KD cells was rarely observed (Figure S1K). Collectively, these results indicate that direct cell-cell interactions between normal and Rpl24-KD cells are required for the induction of cell death, whereas secreted factors alone are not sufficient. We next established MDCK cells inducing knockdown of ribosomal protein small subunit 3 (Rps3), another ribosomal protein, in a tetracycline-dependent manner (Figures S2A and S2B). As with Rpl24 knockdown, knockdown of Rps3 also suppressed both protein synthesis and cell proliferation (Figure S2C and data not shown). We then demonstrated that the comparable loser phenotype was observed for Rps3-knockdown cells (Figure S2D), indicating that loss of ribosomal protein function can induce cell competition in the mammalian cell culture system.

Video S1. Cell competition-mediated cell death of Rpl24-knockdown cells surrounded by normal cells (higher magnification), related to Figure 1 Figure 1E shows a cropped image from this movie. Normal MDCK cells were co-cultured with MDCK-pTR Rpl24-shRNA1 cells at a ratio of 10:1 in the presence of tetracycline. Images were captured at 30-min intervals. Scale bars, 10 μm.

Video S2. Cell competition-mediated cell death of Rpl24-knockdown cells surrounded by normal cells (lower magnification), related to Figure 1 Normal MDCK cells were co-cultured with MDCK-pTR Rpl24-shRNA1 cells at a ratio of 10:1 in the presence of tetracycline. Images were captured at 30-min intervals. Scale bars, 10 μm.

Video S3. No cell death without Rpl24 knockdown, related to Figure 1 Normal MDCK cells were co-cultured with MDCK-pTR Rpl24-shRNA1 cells at a ratio of 10:1 in the absence of tetracycline. Images were captured at 30-min intervals. Scale bars, 10 μm.

### Rpl24-knockdown cells undergo p53-mediated apoptosis during cell competition with surrounding normal cells

**Figure 2:**
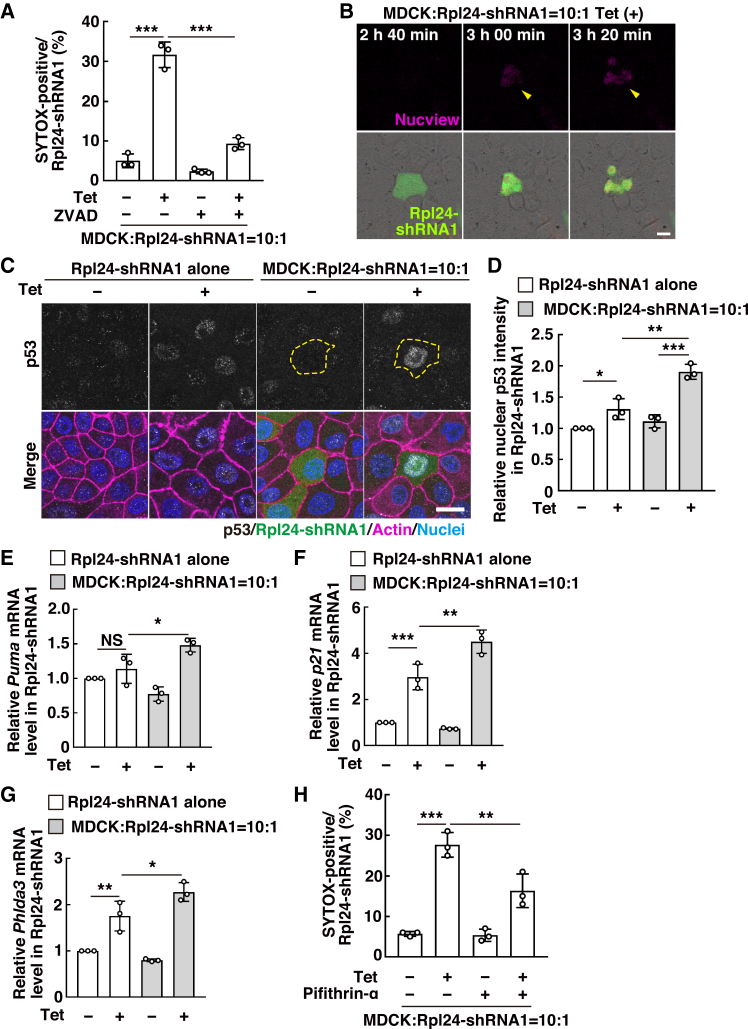
p53-induced apoptosis of Rpl24-knockdown cells during cell competition with surrounding normal cells (A) Effect of Z-VAD-FMK on cell death of Rpl24-shRNA cells. MDCK-pTR Rpl24-shRNA1 cells were co-cultured with normal MDCK cells with or without tetracycline and/or Z-VAD-FMK (ZVAD). Cell death was analyzed with SYTOX-dye. Data are mean ± SD from three independent experiments (*n* = 100 cells for each experiment). ∗∗∗*p* < 0.001 (one-way ANOVA with Tukey’s test). (B) Activation of caspase-3 in dying Rpl24-knockdown cells surrounded by normal cells. MDCK-pTR Rpl24-shRNA1 cells (GFP-positive) were co-cultured with normal MDCK cells with tetracycline, followed by incubation with NucView to analyze the activation of caspase-3. The yellow arrowheads indicate a NucView-positive cell. (C and D) Immunofluorescence analysis of p53. MDCK-pTR Rpl24-shRNA1 cells were cultured alone or co-cultured with normal MDCK cells with or without tetracycline under confluent conditions. Cells were then stained with anti-p53 antibody (white), Alexa Fluor 568-phalloidin (magenta), and Hoechst (blue). The yellow dashed line indicates an Rpl24-shRNA1 cell. Note that, GFP signals are not shown in “Rpl24-shRNA1 alone” to clearly delineate the contour of each cell. (D) Quantification of fluorescence intensity of nuclear p53. Values are expressed as a ratio relative to Rpl24-shRNA1 alone (Tet -). Data are mean ± SD from three independent experiments (*n* = 50 cells for each experiment). ∗*p* < 0.05, ∗∗*p* < 0.01, ∗∗∗*p* < 0.001 (one-way ANOVA with Tukey’s test). (E–G) qPCR analysis for the expression of p53-downstream targets in Rpl24-shRNA cells. MDCK-pTR Rpl24-shRNA1 cells were cultured alone or co-cultured with normal MDCK cells with or without tetracycline under confluent conditions. Rpl24-shRNA1 cells were then collected by FACS, and the RNAs were examined by qPCR. Values are expressed as a ratio relative to Rpl24-shRNA1 alone (Tet -). Data are mean ± SD from three independent experiments. ∗*p* < 0.05, ∗∗*p* < 0.01, ∗∗∗*p* < 0.001, NS, not significant (one-way ANOVA with Tukey’s test). (H) Effect of p53 inhibitor on cell competition-mediated cell death of Rpl24-shRNA cells. MDCK-pTR Rpl24-shRNA1 cells were co-cultured with normal MDCK cells with or without tetracycline and/or pifithrin-α. Cell death was analyzed with SYTOX dye. Data are mean ± SD from three independent experiments (*n* = 100 cells for each experiment). ∗∗*p* < 0.01, ∗∗∗*p* < 0.001 (one-way ANOVA with Tukey’s test). (B and C) Scale bars, 10 μm. See also Figure S3.

We next explored the underlying molecular mechanism of cell death in Rpl24-knockdown cells. Treatment with the caspase inhibitor Z-VAD-FMK profoundly suppressed the SYTOX-positive ratio of Rpl24-knockdown cells that were co-cultured with normal cells (Figure 2A). In addition, when Rpl24-knockdown cells were apically extruded or fragmented, they became positive for the fluorogenic caspase substrate Nucview, an indicator for caspase-3 activation (Figure 2B), indicating that Rpl24-knockdown cells underwent apoptosis. In previous studies, we have demonstrated that Mahjong-knockdown or Scribble-knockdown cells died in a c-Jun N-terminal kinase (JNK)- or p38 mitogen-activated protein kinase (MAPK)-dependent manner, respectively, upon cell competition with surrounding normal cells.^16^^,^^17^ However, addition of the JNK inhibitor SP600125 or the p38 MAPK inhibitor SB202190 did not suppress the cell death in Rpl24-knockdown cells (Figures S3A and S3B). In *Drosophila*, autophagy has been reported to induce the loser phenotype in *Minute* cell competition.^18^ We therefore asked whether autophagy is activated in Rpl24-knockdown cells during competition with surrounding normal cells. LC3-positive puncta were increased when Rpl24-knockdown cells were surrounded by normal cells, but not when they were cultured alone (Figures S3C and S3D). Because chloroquine blocks the late lysosomal degradation step of autophagy, we next examined its effect on LC3 puncta accumulation. Chloroquine treatment significantly increased the number of LC3-positive puncta in Rpl24-knockdown cells surrounded by normal cells (Figures S3C and S3D), indicating that the process of autophagy (autophagic flux) is enhanced in competing Rpl24-knockdown cells. By contrast, treatment with 3-methyladenine (3-MA), which inhibits an early step of autophagosome formation, reduced the accumulation of LC3-positive puncta (Figure S3E). However, 3-MA did not suppress the frequency of death induction in Rpl24-knockdown cells (Figure S3F), suggesting that, although autophagy is activated during cell competition, apoptotic cell death is not induced by autophagy. We then realized that most Rpl24-knockdown cells had a single, large nucleolus (Figures S3G–S3I), a characteristic phenotype of nucleolar stress, which is often accompanied by the activation of the p53 pathway.^19^^,^^20^ Indeed, nuclear p53 accumulation was frequently observed in Rpl24-knockdown cells in monoculture, and was further enhanced when they were co-cultured with normal cells (Figures 2C and 2D). Furthermore, the expression of p53-downstream targets, *Puma*, *p21*, and *Phlda3* was substantially elevated in Rpl24-knockdown cells (Figures 2E–2G). Moreover, treatment with the p53 inhibitor pifithrin-α, which suppressed the expression of the p53-downstream target, significantly reduced the frequency of cell death in co-cultured Rpl24-knockdown cells, but not in monocultured Rpl24-knockdown cells (Figures 2H, S3J, and S3K). These results suggest that the p53 pathway is involved, at least partly, in the induction of cell competition-mediated apoptosis in Rpl24-knockdown cells; however, because of the partial effect of the p53 inhibitor, it is likely that other mechanism(s) are also involved.

### Rpl24 knockdown induces cell enlargement and reduces cell-surface tension

**Figure 3:**
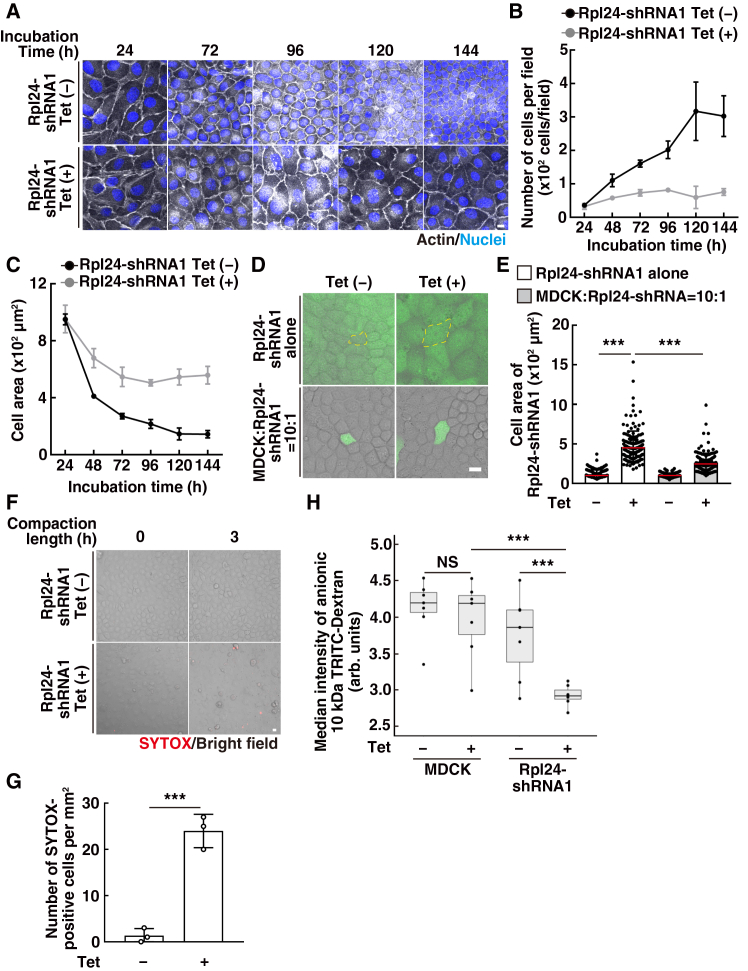
Effect of Rpl24 knockdown on homeostatic cell density, cell area, and cell-surface tension (A–C) Effect of Rpl24 knockdown on homeostatic cell density. MDCK-pTR Rpl24-shRNA1 cells were cultured alone with or without tetracycline for the indicated times. Cells were then stained with Alexa Fluor 647-phalloidin (white) and Hoechst (blue). (B and C) Time-course measurement of cell number (B) and cell area (C). Data are mean ± SD from three independent experiments. Cells within 10 fields (0.21 × 0.21 mm^2^) were analyzed for each experiment. (D and E) Effect of Rpl24 knockdown on cell area. MDCK-pTR Rpl24-shRNA1 cells (GFP-positive) were cultured alone or co-cultured with normal MDCK cells with or without tetracycline under confluent conditions. The yellow dashed line indicates a single Rpl24-shRNA1 cell. (E) Quantification of cell area. Data are mean ± SD from 150 cells from three independent experiments. ∗∗∗*p* < 0.001 (one-way ANOVA with Tukey’s test). (F and G) Effect of Rpl24 knockdown on compaction-mediated cell death. MDCK-pTR Rpl24-shRNA1 cells were cultured alone with or without tetracycline under confluent conditions on the stretchable PDMS membrane. Cell compaction assay was then performed, and cell death was analyzed by time-lapse observation using SYTOX dye. (G) Quantification of cell death under the compacted condition. Data are mean ± SD from three independent experiments. ∗∗∗*p* < 0.001 (unpaired two-tailed Student’s *t* test). The number of SYTOX-positive cells was counted within three fields (0.64 × 0.64 mm^2^) for each experiment. (H) Effect of Rpl24 knockdown on cell-surface tension by ELASTomics approach. MDCK or MDCK-pTR Rpl24-shRNA1 cells were cultured alone with or without tetracycline under confluent conditions, followed by the ELASTomics procedure. Cell-surface tension was measured by analyzing the fluorescence intensity of intracellularly incorporated TRITC-dextran in FACS-sorted cells. Boxplots showing the per-experiment median fluorescence intensity of anionic 10 kDa TRITC-dextran imported by ELASTomics across independent experiments (*n* = 4,285–28,403 cells per experiment). In the boxplots, the center line indicates the median, whiskers indicate the minimum and maximum values excluding outliers, and dots indicate each experiment (*N* = 7). ∗∗∗*p* < 0.001, NS, not significant (one-way ANOVA with Tukey’s test). (A, D, and F) Scale bars, 10 μm. See also Figure S4.

While repeatedly culturing MDCK-pTR Rpl24-shRNA cells, we noticed that Rpl24 knockdown influenced the cell area. In cultured epithelial monolayers, even after reaching 100% confluency, cells continue to proliferate for a while and eventually stop dividing when they reach a maximal cell density, termed homeostatic cell density.^21^^,^^22^ We found that when MDCK-pTR Rpl24-shRNA cells were cultured in the presence of tetracycline, they stopped proliferating at a lower cell density, compared with those cultured without tetracycline (Figures 3A and 3B), indicating that Rpl24-knockdown cells exhibit lower homeostatic cell density. Accordingly, the area of Rpl24-knockdown cells remained large even after long incubation (Figure 3C). In addition, when Rpl24-knockdown cells were co-cultured with normal cells, the surrounding normal cells continued to proliferate after Rpl24-knockdown cells entered growth arrest, inducing compaction and a decrease in the cell area of Rpl24-knockdown cells (Figures 3D and 3E). Treatment with pifithrin-α did not affect the cell area of Rpl24-knockdown cells (Figure S4A), indicating that the p53 pathway is not involved in this biophysical property of Rpl24-knockdown cells. We next examined how Rpl24-knockdown cells respond to the compacted condition. MDCK-pTR Rpl24-shRNA cells were cultured on the stretchable polydimethylsiloxane (PDMS) membrane in the absence or presence of tetracycline, and 8% compaction was applied. When Rpl24 was knocked down, the externally imposed compaction frequently induced cell death-mediated cell extrusion (Figures 3F and 3G), suggesting that Rpl24 knockdown induces higher sensitivity to cell compaction. In addition, p53-downstream target gene expression was not substantially elevated in Rpl24-knockdown cells under the compacted condition (Figures S4B–S4D). Moreover, to measure cell-surface tension, we utilized an ELASTomics approach in which nanopore electroporation causes the incorporation of TRITC-CM-dextran into the analyzed cells in a cell-surface tension-dependent manner.^23^^,^^24^ The number of molecules imported through these nanopores can then be used as a readout of cell-surface tension. In previous studies, DNA-tagged dextran (DTD) and fluorescein isothiocyanate (FITC)-bovine serum albumin (BSA) were used as cargo molecules for this measurement, whereas in the present study, we used anionic TRITC-dextran (10 kDa) and confirmed that this approach could detect the actin-depolymerization inhibitor Cytochalasin D (CytoD)-induced decrease in cell-surface tension (Figure S4E). We then found that much smaller amounts of TRITC-CM-dextran were delivered into Rpl24-knockdown cells, demonstrating the reduction in the cell-surface tension (Figure 3H). Hence, knockdown of Rpl24 leads to biophysical changes in homeostatic cell density and cell-surface tension.

### Proteostatic defect drives the biophysical changes and the loser phenotype in Rpl24-knockdown cells

**Figure 4:**
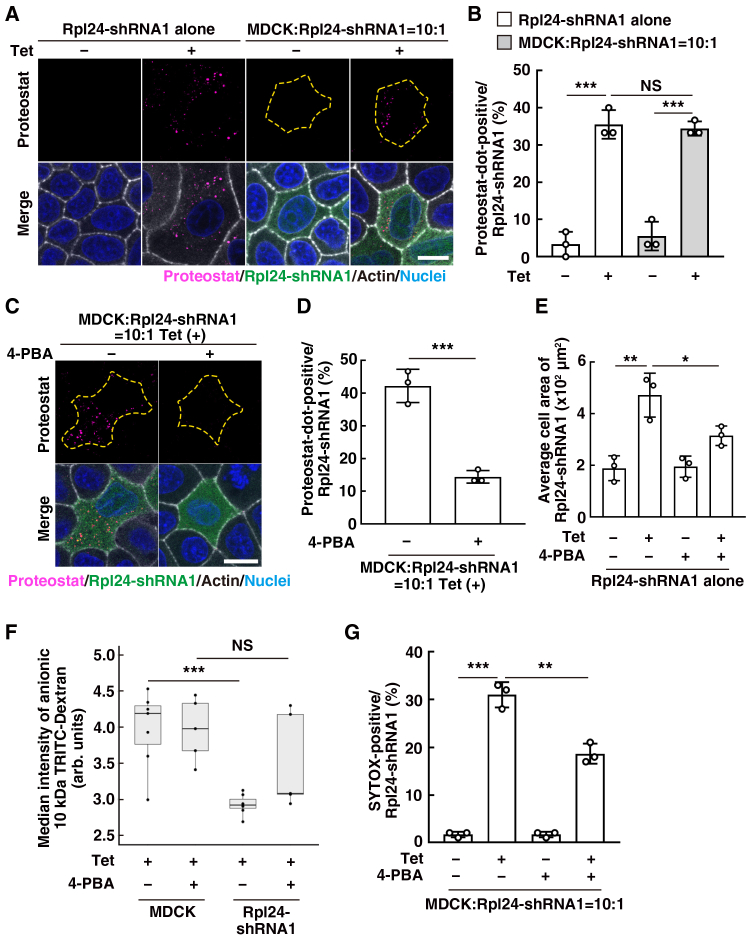
Involvement of proteostasis defect in the biophysical alterations and the loser phenotype of Rpl24-knockdown cells (A and B) Effect of Rpl24 knockdown on the formation of protein aggregates. MDCK-pTR Rpl24-shRNA1 (GFP-positive) cells were cultured alone or co-cultured with normal MDCK cells with or without tetracycline under confluent conditions. Cells were then stained with Proteostat (magenta), Alexa Fluor 647-phalloidin (white), and Hoechst (blue). The yellow dashed line indicates an Rpl24-shRNA1 cell. Note that GFP signals are not shown in “Rpl24-shRNA1 alone” to clearly delineate the contour of each cell. (B) Quantification of cells with Proteostat-positive protein aggregates. Data are mean ± SD from three independent experiments (*n* = 30 cells for each experiment). ∗∗∗*p* < 0.001, NS, not significant (one-way ANOVA with Tukey’s test). (C and D) Effect of 4-PBA on protein aggregates in Rpl24-knockdown cells. MDCK-pTR Rpl24-shRNA1 cells were co-cultured with normal MDCK cells with tetracycline in the presence or absence of 4-PBA. Cells were then stained with Proteostat (magenta), Alexa Fluor 647-phalloidin (white), and Hoechst (blue). The yellow dashed line indicates an Rpl24-shRNA1 cell. (D) Quantification of cells with Proteostat-positive protein aggregates. Data are mean ± SD from three independent experiments (*n* = 30 cells for each experiment). ∗∗∗*p* < 0.001 (unpaired two-tailed Student’s *t* test t). (E) Effect of 4-PBA on cell area of Rpl24-knockdown cells. MDCK-pTR Rpl24-shRNA1 cells were cultured alone with or without tetracycline and/or 4-PBA under confluent conditions. Data are mean ± SD from three independent experiments (*n* = 50 cells for each experiment). ∗*p* < 0.05, ∗∗*p* < 0.01 (one-way ANOVA with Tukey’s test). (F) Effect of 4-PBA on cell-surface tension of Rpl24-knockdown cells. MDCK or MDCK-pTR Rpl24-shRNA1 cells were cultured alone with tetracycline in the absence or presence of 4-PBA under confluent conditions, followed by the ELASTomics procedure. Boxplots showing the per-experiment median fluorescence intensity of anionic 10 kDa TRITC-dextran imported by ELASTomics across independent experiments (*n* = 4,195–27,162 cells per experiment). In the boxplots, the center line indicates the median, whiskers indicate the minimum and maximum values excluding outliers, and dots indicate each experiment. MDCK Tet (+)/4-PBA (−) and Rpl24-shRNA1 Tet (+)/4-PBA (−) were the same experimental samples as those shown in Figure 3H. *N* = 7, 5, 7, 5. ∗∗∗*p* < 0.001, NS, not significant (one-way ANOVA with Tukey’s test). (G) Effect of 4-PBA on cell competition-mediated cell death of Rpl24-shRNA cells. MDCK-pTR Rpl24-shRNA1 cells were co-cultured with normal MDCK cells with or without tetracycline and/or 4-PBA. Cell death was analyzed with SYTOX dye. Data are mean ± SD from three independent experiments (*n* = 100 cells for each experiment). ∗∗*p* < 0.01, ∗∗∗*p* < 0.001 (one-way ANOVA with Tukey’s test). (A and C) Scale bars, 10 μm. See also Figure S5.

Previous studies in *Drosophila* have demonstrated that, in *Minute*-mutant cells, proteostatic processes become defective, and protein aggregates accumulate, thereby driving loser status in cell competition.^11^^,^^12^ We then examined the accumulation of protein aggregates using Proteostat, a fluorescent probe that labels protein aggregates. We found that Proteostat fluorescence-positive dots were diffusely present throughout the cytoplasm in Rpl24-knockdown cells that were cultured alone or co-cultured with normal cells, whereas the Proteostat dots were not frequently observed in Rpl24-shRNA cells cultured in the absence of tetracycline (Figures 4A and 4B), indicating that Rpl24 knockdown impairs proteostasis. 4-Phenylbutyric acid (4-PBA) is a widely used chemical chaperone that improves cellular proteostasis by facilitating protein folding and reducing misfolded protein aggregates in the endoplasmic reticulum and other intracellular compartments.^25^ We first confirmed that 4-PBA treatment suppressed the accumulation of Proteostat-positive aggregates in Rpl24-knockdown cells (Figures 4C and 4D). We then found that 4-PBA treatment partially reversed the changes in homeostatic cell density and cell-surface tension in Rpl24-knockdown cells (Figures 4E and 4F), indicating that these Rpl24 knockdown-mediated biophysical alterations are attributed, at least in part, to proteostatic defects. Furthermore, 4-PBA treatment significantly reduced the frequency of cell death induction in Rpl24-knockdown cells co-cultured with normal cells (Figure 4G), but did not suppress the expression of p53 target genes (Figures S5A–S5C). Moreover, 4-PBA treatment suppressed the compaction-mediated death of Rpl24-knockdown cells (Figure S5D). Together with the previous studies demonstrating that homeostatic density or cell-surface tension can influence the occurrence of cell competition/cell extrusion,^26^^,^^27^ these results suggest that Rpl24 knockdown-induced proteostatic defects influence cellular biophysical properties and sensitivity to cell compaction, thereby driving a loser phenotype in cell competition, independently of p53 activity.

## Discussion

In this study, using a mammalian cell culture system, we demonstrate that Rpl24-knockdown cells undergo apoptosis and are eliminated from an epithelial layer through cell competition with surrounding normal cells. Our data support a model in which two parallel, independent inputs—(i) nucleolar stress-associated p53 activation and (ii) proteostatic defect-driven biophysical alterations—cooperate to promote the loser fate of Rpl24-knockdown cells during cell competition. Pharmacological inhibition of p53 reduces, but does not fully abrogate, cell competition-mediated elimination of Rpl24-knockdown cells, whereas the chemical chaperone 4-PBA suppresses cell death without attenuating p53 target gene induction, consistent with the notion that these pathways act at least in part independently.

In monocultured Rpl24-knockdown cells, activity of the p53 pathway is moderately elevated. Rpl24-knockdown cells also display a single, large nucleolus, a characteristic phenotype of nucleolar stress, which is often accompanied by the activation of the p53 pathway.^19^^,^^20^ It is likely that Rpl24 knockdown causes imbalanced ribosomal protein availability and impaired ribosome biogenesis, leading to nucleolar stress and p53 activation. Despite this moderate p53 activation, cell death does not frequently occur in monocultured Rpl24-knockdown cells. Importantly, stronger activation of p53 is observed when Rpl24-knockdown cells are co-cultured with normal cells. Thus, Rpl24-knockdown cells exhibit a cell-intrinsic upregulation of p53, which is further enhanced by the presence of surrounding normal cells in a non-cell-autonomous manner, thereby inducing apoptosis. Intriguingly, a comparable phenomenon has also been described in *Drosophila Minute* cell competition. When *Minute*-mutant cells are surrounded by wild-type cells, they undergo apoptosis through JNK activation. Importantly, cell-autonomous JNK activation has also been observed in *Minute*-mutant cells in *Minute*-mutant larvae, in which all epithelial cells carry *Minute* mutations.^28^ This suggests that *Minute*-mutant cells intrinsically exhibit elevated JNK signaling, whereas additional competition-dependent inputs are likely required for efficient apoptosis. These observations raise the possibility that a similar principle may operate in mammalian cell competition, where cell-intrinsic stress responses in loser cells are further reinforced by signals from surrounding winner cells.

In *Drosophila*, previous studies have demonstrated that increased proteotoxic stress can induce loser status in *Minute*-mutant cells.^11^^,^^12^ However, it remained unclear whether the comparable phenomenon also occurs in mammals and how proteotoxic stress triggers cell competition. We demonstrate that, in Rpl24-knockdown cells, protein aggregates accumulate in the cytoplasm, indicating that Rpl24 knockdown disrupts proper regulation of protein quality control. Although autophagic flux is elevated when Rpl24-knockdown cells are surrounded by normal cells, pharmacological blockade at the early stage of autophagy does not suppress apoptosis, arguing that autophagy is a correlated stress response rather than a primary execution mechanism. A previous study demonstrated that imbalances in the expression of ribosomal proteins lead to the rapid aggregation of newly synthesized “orphan” ribosomal proteins.^29^ Indeed, in Rpl24-knockdown cells, we found that other ribosomal proteins such as Rpl5 and Rps3 often form cytoplasmic dotty structures (Figures S5E and S5F). These results imply that Rpl24 knockdown leads to a failure of ribosome assembly and that massive amounts of non-incorporated, free ribosomal proteins form protein aggregates, thereby disrupting cellular proteostasis. Rpl24-knockdown cells also exhibit morphological and physical changes including lower homeostatic density, increased cell area, and decreased cell-surface tension. Previous mathematical modeling and biophysical studies have shown that when cells with lower homeostatic density or cell-surface tension appear within an epithelial monolayer, they are eliminated from the cellular society through a mechanically driven process.^26^^,^^27^ Consistent with these predictions, Rpl24-knockdown cells, which exhibit these biophysical traits, are eliminated as losers during cell competition with surrounding normal cells. Importantly, we demonstrate that treatment with the chemical chaperone 4-PBA diminishes these physical alterations, indicating that proteostatic defects profoundly influence cellular biophysical properties, thereby contributing, at least in part, to the loser phenotype of cell competition. Moreover, perturbation of proteostasis by the proteasome inhibitor MG-132 phenocopies key morphological changes such as increased cell area, which is rescued by 4-PBA treatment (Figures S5G–S5I), supporting the notion that proteostasis disruption per se is sufficient to drive the biophysical remodeling processes.

A previous study demonstrated that, when Scribble-knockdown cells are co-cultured with normal cells, the surrounding normal cells move toward the Scribble-knockdown cells and mechanically compress them, leading to their apoptotic elimination; this process is termed mechanical cell competition.^30^ Mechanical cell competition and the cell competition described in this study share the feature that compressive forces contribute to apoptosis of loser cells with a lower homeostatic density and increased sensitivity to cell compaction. However, there also appears to be a difference in the underlying mechanisms. In Scribble-knockdown-mediated mechanical cell competition, p38MAPK acts upstream of p53, and activated p53 influences homeostatic cell density. By contrast, in Rpl24-knockdown-mediated cell competition, inhibition of p38MAPK did not suppress the cell death in Rpl24-knockdown cells, and p53 inhibition did not alter homeostatic cell density, suggesting that p53-dependent regulation of homeostatic density is not a universal feature of loser cells. It remains to be determined how prevalently these physical properties, including lower homeostatic density and increased sensitivity to cell compaction, contribute to the loser phenotype in various contexts of cell competition.

In the present study, ELASTomics analysis showed that cell-surface tension is reduced in Rpl24-knockdown cells. Intriguingly, recent studies have suggested that lower stiffness or higher compressibility can contribute to the loser status during cell competition,^26^^,^^31^ although these properties are distinct from cell-surface tension. Thus, while the mechanistic link between reduced cell-surface tension and competitive elimination remains to be clarified, our findings support the notion that altered biophysical properties are involved in cell competition and provide a basis for future studies to define how distinct mechanical properties influence loser cell behavior.

Recent studies present several lines of evidence that dysregulated protein homeostasis plays a crucial role in driving cell competition.^5^ An important future direction will be to determine how prevalently proteostasis defects in loser cells are converted into biophysical changes (e.g., cell area, homeostatic density, cell-surface tension) and how winner cells sense these alterations to trigger the elimination of loser cells. Identifying the mechanosensing and downstream effector modules in winner cells will provide a mechanistic link between intracellular proteostasis failure and tissue-level surveillance.

### Limitations of the study

It remains unclear whether and how neighboring normal cells non-cell-autonomously enhance p53 pathway activation and induce additional cell competition-dependent signals in Rpl24-knockdown cells. We show that autophagy is activated during cell competition, but does not play a key role in the induction of apoptosis. Because externally imposed compaction does not substantially elevate p53 activity (Figures S4B–S4D), the non-cell-autonomous p53 activation in co-cultures is unlikely to be induced by compaction alone, raising the possibility that biochemical signaling from surrounding normal cells also contributes to this process. This mechanism remains to be elucidated in future studies.

## Resource availability

### Lead contact

Further information and requests for resources and reagents should be directed to and will be fulfilled by the lead contact, Yasuyuki Fujita (fujita@monc.med.kyoto-u.ac.jp).

### Materials availability

Plasmids or cell lines generated in this study will be made available on reasonable request, but we may require payment and/or a completed Materials Transfer Agreement if there is potential for commercial application.

### Data and code availability

• Data reported in this paper will be shared by the lead contact upon request.
• This paper does not report the original code.
• Any additional information required to reanalyze the data reported in this paper is available from the lead contact upon request.

## Acknowledgments

We thank Drs. T. Igaki and Y. Tamori for valuable discussions on *Drosophila Minute* cell competition. We acknowledge support from 10.13039/501100001691Japan Society for the Promotion of Science (JSPS) Grant-in-Aid for Scientific Research on Transformative Research 21H05285A01, Grant-in-Aid for Scientific Research (S) 21H05039, 10.13039/501100002241Japan Science and Technology Agency (JST) (Moonshot R&D: grant no. JPMJPS2022), and the 10.13039/100007449Takeda Science Foundation (to Y.F.) and JST (Kyoto University Division of Graduate Studies, Support for Pioneering Research Initiated by the Next Generation Program) (to W.S.).

## Author contributions

W.S. designed experiments and generated most of the data. M.Y., K.K., N.S., J.W., Z. D., and Su.I. assisted with experiments. A.S. and H.S. performed and analyzed ELASTomics. Se.I. and H.H. assisted with the cell compaction assay. Y.F. conceived and designed the study. The manuscript was written by W.S. and Y.F. with assistance from the other authors.

## Declaration of interests

The authors declare no competing interests.

## Declaration of generative AI and AI-assisted technologies in the writing process

During the preparation of this work, the authors used “ChatGPT thinking” for proofreading. After using this tool/service, the authors reviewed and edited the content as needed and take full responsibility for the content of the published article.

## STAR★Methods

### Key resources table

**Table:** 

REAGENT or RESOURCE	SOURCE	IDENTIFIER
**Antibodies**		

Rabbit anti-RPL24	Invitrogen	Cat# PA5-30157; RRID: AB_2547631
Mouse anti-phosphor-histone H3 (Ser10) (6G3)	Cell Signaling Technology	Cat# 9706; RRID: AB_331748
Rabbit anti-p53	Cell Signaling Technology	Cat# 9282; RRID: AB_331476
Mouse anti-RPS3 (C-7)	Santa Cruz	Cat# sc-376008; RRID: AB_10991105
Rabbit anti-RPL5	Abcam	Cat# AB137617; RRID: AB_2924679
Mouse anti-Nucleophosmin1 (FC82291)	Abcam	Cat# AB10530; RRID: AB_297271
Mouse anti-GAPDH	Millipore	Cat# MAB374; RRID: AB_2107445
Horseradish peroxidase-conjugated AffiniPure anti-mouse IgG	Jackson ImmunoResearch	Cat# 715-035-151
Horseradish peroxidase-conjugated AffiniPure anti-rabbit IgG	Jackson ImmunoResearch	Cat# 711-035-152
Alexa Fluor 568-conjugated phalloidin	Life Technologies	Cat# A12380
Alexa Fluor 647-conjugated phalloidin	Life Technologies	Cat# A22287
Alexa Fluor 647 Anti-mouse	Life Technologies	Cat# A31571
Alexa Fluor 647 Anti-rabbit	Life Technologies	Cat# A31573

**Chemicals, peptides, and recombinant proteins**		

Z-VAD-FMK	Calbiochem	Cat# 627610
Pifithrin-a	Sigma-Aldrich	Cat# P4359
SP600125	Sigma-Aldrich	Cat# S5567
SB202190	Calbiochem	Cat# 559397
Chloroquine	Sigma-Aldrich	Cat# C6628
3-Methyladenine	Calbiochem	Cat# 189490
4-Phenylbutyric acid	Sigma-Aldrich	Cat# P21005
Blasticidin	InvivoGen	Cat# ant-bl
G418 (Geneticin)	GIBCO	Cat# 10131027
Puromycin	Sigma-Aldrich	Cat# P8833
Tetracycline	Sigma-Aldrich	Cat# T7660
Trizol	Thermo Fisher Scientific	Cat# 15596026
Fibronectin	Sigma-Aldrich	Cat# F1141
Hoechst 33342	Life Technologies	Cat# H3570
Fibronectin	Corning	Cat# 356008
Pluronic F-127	Sigma-Aldrich	Cat# P2443-250G
Tetramethylrhodamine-labeled anionic dextran (10 kDa)	Thermo Fisher Scientific	Cat# D1868
Trypsin	Nacalai Tesque	Cat# 35555-54
Cytochalasin D	Cayman Chemical Company	Cat# 111330
Calcein AM	Thermo Fisher Scientific	Cat# C3099

**Critical commercial assays**		

Type 1 collagen (Cellmatrix Type 1-A)	Nitta Gelatin	N/A
MycoAlart	Lonza	Cat# LT07-118
Nucleofector 2b Kit L	Lonza	Cat# VACA-1005
Click-iT™ Plus OPP Alexa Fluor™ 647 Protein Synthesis Assay Kit	Invitrogen	Cat# C10458
Click-iT™ EdU Cell Proliferation Kit for Imaging, Alexa Fluor™ 647 dye	Invitrogen	Cat# C10340
SYTOX Orange Nucleic Acid Stain	Thermo Fisher Scientific	Cat# S11368
Nucview 530 Caspase-3 substrate	Biotium	Cat# 10406
GeneAce SYBR qPCR Mix	NIPPON GENE	Cat# 319-07683
RNeasy Micro Kit	Qiagen	Cat# 74004
PROTEOSTAT^(R)^ Aggresome detection kit	Enzo Life Science	Cat# ENZ-51035-K100
Gel-Film	Gel-Pak	Cat# PF-60x60-0065-X4
Polydimethylsiloxane slab	Dow Corning	Cat# SILPOT^TM^ 184 W/C
Track-etched membrane (pore size: 100 nm)	Merck	Cat# VCTP04700
pluriStrainer-Mini	pluriSelect	Cat# 43-10040

**Experimental models: Cell lines**		

MDCK	Dr. W. Birchmeier	N/A

**Oligonucleotides**		

See Table S1	N/A	N/A

**Recombinant DNA**		

pPB RFP-LC3	Akter et al.^32^	N/A
pPB CMV-MCS-EF1a-Puro Vector	System Biosciences	Cat# PB510B-1
pPB CMV-RFP-LC3-EF1a-Puro	This paper	N/A

**Software and algorithms**		

ImageJ	NIH Image	https://imagej.net/ij/download.html
Metamorph	Molecular Devices	https://www.moleculardevices.co.jp/systems/metamorph-microscopy-automation-and-image-analysis-software
GraphPad Prism 7	GraphPad Software	https://www.mdf-soft.com/products/graphpad_prism8.html
FlowJo	BD Biosciences	https://www.bdbiosciences.com/ja-jp/products/software/flowjo-software?tab=flowJo-v11-software

**Other**		

39-mm silicone chamber	Dr. S. Ishihara	N/A
42-mm steel-ring	Dr. S. Ishihara	N/A

### Experimental model and study participant details

#### Cell line

MDCK cells were used in this study. The parental MDCK cells were a gift from W. Birchmeier (MDC, Berlin). Cells were cultured in Dulbecco’s modified Eagle’s medium (DMEM) supplemented with 10% fetal bovine serum (FBS) (Sigma-Aldrich), 1% penicillin/streptomycin (Life Technologies), and 1% GlutaMax (Life Technologies) in a humidified 5% CO_2_ incubator at 37°C. Mycoplasma contamination was regularly tested for all cell lines using MycoAlert Mycoplasma Detection kit (Lonza).

### Method details

#### Antibodies and materials

Rabbit anti-RPL24 (#PA5-30157) antibody was purchased from Invitrogen. Mouse anti-phospho-histone H3 (Ser10) (6G3) (#9706) and rabbit anti-p53 (#9282S) antibodies were from Cell Signaling Technology. Mouse anti-RPS3 (C-7) (#sc-376008) antibody was from Santa Cruz. Rabbit anti-RPL5 (#AB137617) and mouse anti-Nucleophosmin1 (FC82291) (#AB10530) antibodies were from Abcam. Mouse anti-GAPDH (#MAB374) antibody was from Millipore. Alexa Fluor 568- and 647-conjugated phalloidin (Life Technologies) were used at 1.0 unit/mL. Alexa Fluor 647-conjugated secondary antibody was from Life Technologies. Hoechst 33342 (Life Technologies) was used at a dilution of 1:5,000. The inhibitors Z-VAD-FMK (final concentration, 50 μM), SB202190 (10 μM), 3-MA (10 mM), and MG-132 (10 μM) were from Calbiochem. SP600125 (10 μM), chloroquine (40 μM), pifithrin-α (5 μM), and 4-PBA (1 mM) were from Sigma-Aldrich. Type I collagen (Cellmatrix Type I-A) was obtained from Nitta Gelatin and was neutralized on ice according to the manufacturer’s instructions. The Click-iT™ Plus OPP Alexa Fluor™ 647 Protein Synthesis Assay Kit (C10458) was obtained from Invitrogen to stain nascent protein. The Click-iT™ EdU Cell Proliferation Kit, Alexa Fluor™ 647 dye (C10340) was obtained from Invitrogen to stain the entry into S phase in cell cycle. SYTOX™ Orange Nucleic Acid Stain was obtained from Invitrogen to stain dead cells. NucView^TM^ 530 caspase-3 substrate was obtained from Biotium to stain activated caspase-3. PROTEOSTAT Aggresome detection kit (ENZ-51035-K100) was obtained from Enzo Life Science to stain protein aggregates in cells.

#### Establishment of cell lines and cell culture

MDCK cells stably expressing Rpl24-shRNA1, Rpl24-shRNA2, or Rps3-shRNA1 in a tetracycline-inducible manner were established as follows. Rpl24-shRNA1, Rpl24-shRNA2, or Rps3-shRNA1 oligonucleotides were cloned into BglII/XhoI sites of pSUPERIOR.neo+gfp (Oligoengine), and MDCK-pTR cells were transfected with the respective plasmid by nucleofection using Nucleofector 2b (Lonza). The transfected cells were selected in the medium containing 5 μg/mL of blasticidin (Invitrogen) and 800 μg/mL of Geneticin (G418; Gibco). To maintain the cell clones, the medium containing 500 ng/mL of blasticidin and 80 μg/mL of G418 was used. All sequences of shRNA oligonucleotides are shown in Table S1. To establish MDCK-pTR Rpl24-shRNA1 RFP-LC3 cells, the RFP-LC3 sequence from the pPB RFP-LC3 plasmid,^32^ a kind gift from Dr. S. Kon (Tokyo University of Science, Tokyo), was excised using NheI/BamHI and subcloned into the pPB-CMV-MCS-EF1α-Puro vector (System Biosciences). MDCK-pTR Rpl24-shRNA1 cells were then transfected with pPB CMV-RFP-LC3-EF1α-Puro by nucleofection, followed by selection in the medium containing 5 μg/mL of blasticidin, 800 μg/mL of G418, and 500 ng/mL of puromycin (Sigma-Aldrich). To maintain the cell clones, the medium containing 500 ng/mL of blasticidin, 80 μg/mL of G418, and 50 ng/mL of puromycin was used. Cells were plated onto collagen-coated coverslips as previously described,^33^ except for Figures 1B, S1C, S1E, S1G, S2B, and S2C where cells were cultured on non-coated coverslips. For tetracycline-inducible MDCK cell lines, 2 μg/mL of tetracycline (Sigma-Aldrich) was used to induce the expression of shRNAs.

#### Immunofluorescence and western blotting

For immunofluorescence analysis, MDCK-pTR Rpl24-shRNA, MDCK-pTR Rps3-shRNA, and MDCK-pTR Rpl24-shRNA1 mRFP-LC3 cells were pre-treated with 2 μg/mL of tetracycline for 48 h to induce the expression of the respective shRNA, except for Proteostat analysis where the tetracycline pre-treatment was performed for 24 h. Cells were then plated onto coverslips in 12 well-plate as previously described.^33^ For single cell culture, 5.0 x 10^5^ cells were plated, except for Figures 1B, S1E, S1G, S3G, S5G, and S5H where 5.6 x 10^4^ cells were plated and for Figure 4A where 3.0 x 10^5^ cells were plated. For mix cell culture, 4.5 x 10^5^ normal cells and 4.5 x 10^4^ shRNA-expressing cells were plated, except for Figures 4A and 4C where 2.7 x 10^5^ normal cells and 2.7 x 10^4^ shRNA-expressing cells were plated. The fixation was performed after 24 h of plating, except for Figures 4A and 4C where cells were fixed after 48 h of plating. For Figure 3A, 5.0 x 10^5^ cells were plated, and tetracycline was added after 24 h, followed by fixation at 72, 96, 120, and 144 h. Where indicated, the following inhibitors were added before fixation: Z-VAD-FMK (incubation time, 12 h), 3-MA (18 h), 4-PBA (24 h), MG-132 (6 h). Cells were fixed with 4% paraformaldehyde (PFA) in phosphate-buffered saline (PBS), permeabilized, and blocked as previously described.^34^ All primary and secondary antibodies were used at 1:100 and 1:200, respectively. Immunofluorescence images were captured by Olympus FV1200 or FV3000. Using the Olympus FV10-ASW or FV31S-SW software, around 25 xz-sections of immunofluorescence images of randomly selected fields (1,024 x 1,024 pixels) were analyzed. For Figures 1B, S1C, S1E, S1G, S2B, S2C, S3G, and S5H, images of five randomly selected fields (2,048 x 2,048 pixels) were captured using a phase-contrast microscope through the MetaMorph software (Molecular Devices). For image quantification analysis, using the ImageJ software, we selected a plane with the largest nuclear area, except for Proteostat dots analysis where the section located 1 μm apical to the plane showing the largest nuclear area was selected. For western blotting analysis, 2.0 x 10^5^ cells were plated into a 6-well plate, and western blotting was performed as previously described.^35^ Primary and secondary antibodies were used at 1:1,000. Western blotting data were analyzed using ImageQuant LAS4010 (GE healthcare).

#### Time-lapse observation of cultured cells

During live-cell imaging, cells were incubated in the Leibovitz’s L-15 medium (Gibco) containing 10% FBS. Time-lapse images were captured and analyzed by Nikon confocal microscopy (A1 HD25) with the NIS-Elements software (Nikon). Acquired data were analyzed by the ImageJ software. First, shRNA-expressing cells were pre-treated with 2 μg/mL of tetracycline for 48 h and were then plated on the collagen-coated 8-well cover-glass chamber (IWAKI). For single cell culture, 1.65 x 10^5^ shRNA-expressing cells were plated, while for mix cell culture, 1.5 x 10^5^ normal cells and 1.5 x 10^4^ shRNA-expressing cells were plated. For Figure S1I, 3.75 x 10^4^ normal cells and 3.75 x 10^3^ Rpl24-shRNA1 cells were plated. After 24 h of plating, the occurrence of cell competition was analyzed by 24 h-time-lapse imaging with 30-min intervals except for NucView analysis where the images were captured every 5 min. SYTOX^TM^ Orange Nucleic Acid Stain (0.5 μM) (Invitrogen) or NucView^TM^ 530 Caspase-3 substrate (2 μM) (Biotium) was added 30 min before time-lapse started. Where indicated, the following inhibitors were added at the respective time point (Z-VAD-FMK: -12 h (at 12 h before the time-lapse observation started), SP600125: -20 h, SB202190: 0 h, chloroquine: 0 h, 3-MA: 0 h, pifithrin-α: -18 h, 4-PBA: -6 h). SYTOX-positive cells were counted throughout the 24 h-time-lapse observation using the ImageJ software. For Figure S1K, monocultured Rpl24-shRNA1 cells were incubated on the collagen-coated 8-well cover-glass chamber with the conditioned medium from 24-h co-cultured normal and Rpl24-shRNA1 cells at a ratio of 10:1. For Figures 3D and 3E, the cell area of co-cultured cells with tetracycline treatment was quantified 30 min before showing SYTOX-positive signal, whereas the cell area was quantified at the end time-point of time-lapse observation in the other conditions. For Figure S1J, 3.75 x 10^4^ normal cells and 3.75 x 10^3^ Rpl24-shRNA1 cells were plated. After 48 h, cells were treated with tetracycline for 72 h, followed by time-lapse observation.

#### Quantitative real-time PCR (qPCR)

For quantitative real-time PCR for Figures 2E–2G and S5A–S5C, MDCK-pTR Rpl24-shRNA1 cells were first pre-treated with tetracycline for 48 h. Then, for single cell culture, 5.0 x 10^5^ MDCK-pTR Rpl24-shRNA1 cells were plated in a 12-well plate (Corning), while for mix cell culture, 4.5 x 10^5^ MDCK and 4.5 x 10^4^ MDCK-pTR Rpl24-shRNA1 cells were plated. After 24 h incubation, GFP-positive Rpl24-shRNA1 cells were collected by an analytical flow cytometer (SH800S, SONY). Then, total RNAs were extracted using RNeasy Micro Kit (Qiagen) according to the manufacturer’s instructions and reverse-transcribed using QuantiTect Reverse Transcription (Qiagen). For Figures S4B–S4D, TRIzol (Thermo Fisher Scientific) was used to extract the total RNA instead of RNeasy Micro Kit. qPCR reactions were performed with GeneAce SYBR qPCR Mix (NIPPON GENE) using the StepOne system (Thermo Fisher Scientific). Relative quantification analysis was performed with the comparative CT method (2-ΔΔCT) using GAPDH as a reference gene to normalize data. The primer sequences are shown in Table S1.

#### Cell compaction assay

For cell compaction assay (Figures 3F, 3G, and S5D), MDCK-pTR Rpl24-shRNA1 cells were first pre-treated with tetracycline for 24 h and seeded onto fibronectin-coated 39-mm silicone chamber which pre-stretched with 42-mm steel-ring as previously described.^36^ The steel-ring was then removed after 48 h. For time-lapse observations, the medium was changed to the Leibovitz’s L-15 medium (Gibco) containing 10% FBS and SYTOX before removing the steel-ring. For Figure S5D, 4-PBA was added at 24 h after plating on the chamber. The occurrence of cell death was analyzed with SYTOX-dye by 3 h-time-lapse imaging with 30-min intervals as described above.

#### Measurement of cell-surface tension

We employed the ELASTomics approach to measure cell-surface tension. A custom-made cell culture chamber was used, consisting of a polydimethylsiloxane (PDMS) slab (SILPOT^TM^ 184 W/C, Dow Corning) with a track-etched membrane (VCTP04700, Merck; pore size: 100 nm) at the bottom, which was coated with 10 μg/mL fibronectin (356008, Corning). MDCK cells were cultured in the chamber at 37°C for 1 day. After washing with PBS, the bottom surface of culture chamber was coated with a HEPES-based buffer (20 mM HEPES/NaOH, pH 7.0, 260 mM sucrose) containing 0.1% Pluronic F-127 (P2443-250G, Sigma-Aldrich) to prevent non-specific adsorption. The chamber was then placed on an electrode holder prefilled with 150 μL of the HEPES-based buffer containing 0.5 mg/mL tetramethylrhodamine (TRITC)-labeled anionic dextran (10 kDa; D1868, Thermo Fisher Scientific). After a 3 min incubation, square electrical pulses (12 V, 5 ms, 20 Hz) were applied for 500 cycles using a multifunction generator (WF1982, NF Corporation) and a high-speed bipolar amplifier (HSA42051, NF Corporation). The applied voltage was optimized on the basis of cell viability and dextran uptake efficiency. After a 2 min incubation, PBS in the chamber was replaced with fresh culture medium. Cells were incubated for 30 min at 37°C under 5% CO_2_. Subsequently, cells were detached from the membrane using trypsin (35555-54, Nacalai Tesque), washed twice with PBS, and filtered through a 40 μm pluriStrainer-Mini (43-10040, pluriSelect). Fluorescence intensity of the imported dextran was measured using a BD FACS LSR Fortessa X-20 (BD Biosciences) and analyzed with the FlowJo software (BD Biosciences). Dead cells were excluded from the analysis. For validation experiments of ELASTomics (Figure S4E), MDCK cells were treated with DMSO or Cytochalasin D (4 μM) (111330, Cayman Chemical Company) for 1 h at 37°C. ELASTomics was then performed under conditions largely identical to those described above, except that square electrical pulses (4 V, 5 ms, 20 Hz) were applied instead of 12 V. For fluorescence imaging, cells were stained with Calcein AM (C3099, Thermo Fisher Scientific) after ELASTomics and analyzed using an SP8 confocal microscope (Leica).

### Quantification and statical analysis

#### Statistics analysis

To compare the difference between two groups, unpaired two-tailed Student’s t-tests were conducted. For multiple comparisons, one-way ANOVA with Tukey’s test was performed. *p*-values less than 0.05 were considered to be significant. No statistical method was used to predetermine sample size.
