# Supplementary material for: Proteostatic defect drives biophysical remodeling that triggers cell competition
Source: iScience. 2026 Jul 14;29(8):116804. doi: 10.1016/j.isci.2026.116804 (PMC13382627; doi:10.1016/j.isci.2026.116804)
Supplement: Document S1. Figures S1–S5 and Table S1 [file mmc1.pdf]

iScience, Volume *29*

Supplemental information

Proteostatic defect drives biophysical remodeling that triggers cell competition

Wonjae Song, Mai Yukitake, Kei Kozawa, Nanami Sato, Jiaying Wen, Zehao Dai, Susumu Ishikawa, Akifumi Shiomi, Hirofumi Shintaku, Seiichiro Ishihara, Hisashi Haga, and Yasuyuki Fujita

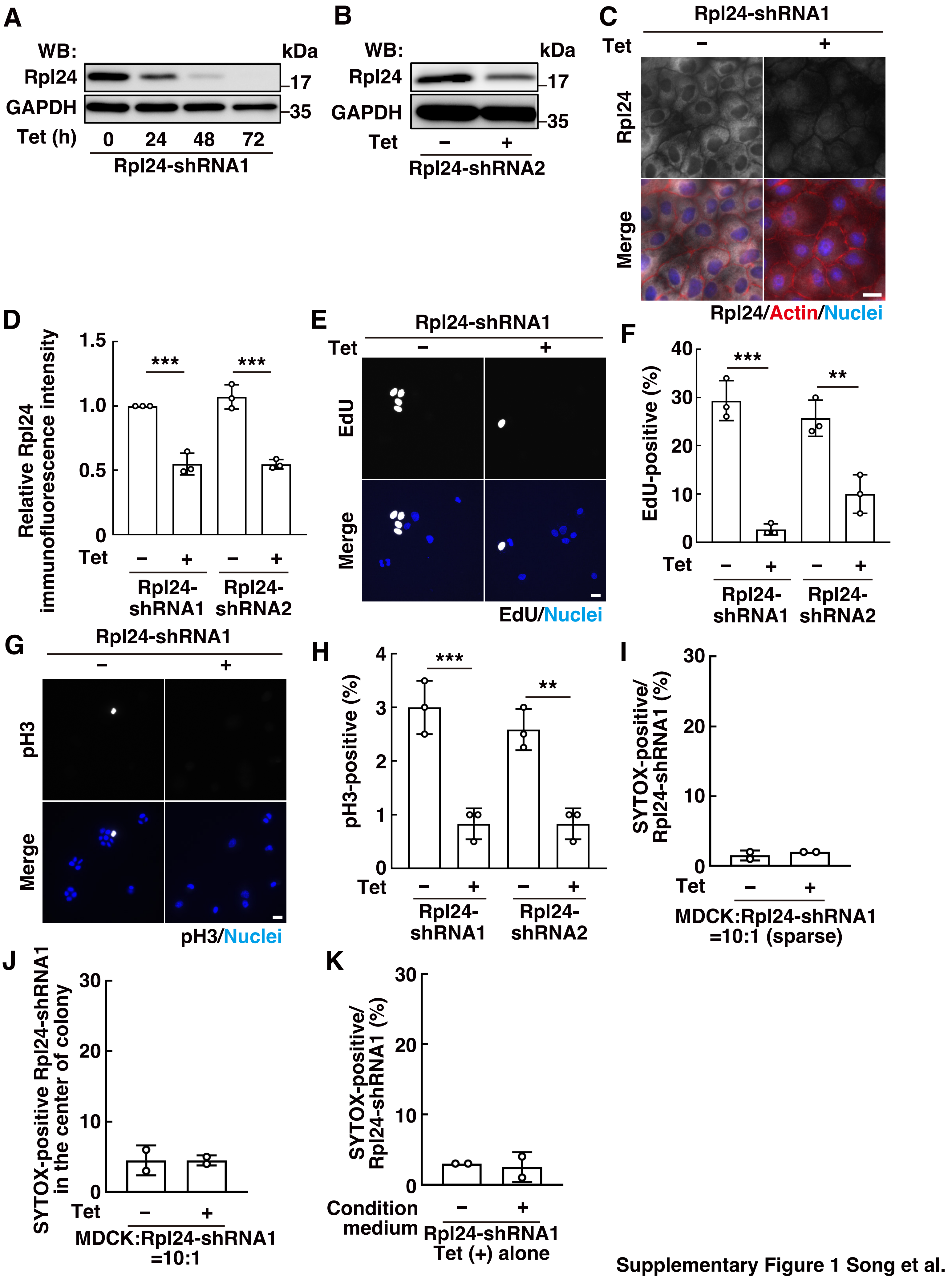

Supplementary Figure 1 Song et al.

Figure S1. Effect of Rpl24 knockdown on cell proliferation, related to Figure 1

(A and B) Rpl24 knockdown induced by tetracycline addition in MDCK-pTR Rpl24-shRNA cells. MDCK-pTR Rpl24-shRNA1 or -shRNA2 cells were incubated with or without tetracycline for the indicated time, and cell lysates were examined by western blotting using anti-Rpl24 and anti-GAPDH antibodies.

(C–H) Effect of Rpl24 knockdown on Rpl24 immunofluorescence (C and D), EdU-positive ratio (E and F), and pH3-positive ratio (G and H). MDCK-pTR Rpl24-shRNA1 cells were cultured with or without tetracycline for 72 h under confluent conditions (C and D) or at low density (E-H). Cells were then stained with anti-Rpl24 antibody, EdU, or anti-pH3 antibody (white), together with Alexa Fluor 568-phalloidin (red) and/or Hoechst (blue). (D) Quantification of Rpl24 immunofluorescence intensity. Values are expressed as a ratio relative to Rpl24-shRNA1 (Tet -). Data are mean ±SD from three independent experiments (n = 50 cells for each experiment). ***p < 0.001 (one-way ANOVA with Tukey's test). (F) Quantification of EdU-positive cells. Data are mean ±SD from three independent experiments (n = 50 cells for each experiment). **p < 0.01, ***p < 0.001 (one-way ANOVA with Tukey's test). (H) Quantification of pH3-positive cells. Data are mean ±SD from three independent experiments (n = 200 cells for each experiment). **p < 0.01, ***p < 0.001 (one-way ANOVA with Tukey's test).

(I) Quantification of cell death of Rpl24-knockdown cells co-cultured with normal cells under sparse conditions. MDCK-pTR Rpl24-shRNA1 cells were co-cultured with normal MDCK cells with or without tetracycline at low density. Cell death was analyzed with SYTOX-dye. Data are mean ±SD from two independent experiments (n = 25 cells for each experiment).

(J) Quantification of cell death of Rpl24-knockdown cells located in the center of colonies that were surrounded by normal cells. MDCK-pTR Rpl24-shRNA1 cells were co-cultured with normal MDCK cells for a longer period (48 h) and incubated with or without tetracycline. Cell death was analyzed with SYTOX-dye. Data are mean ±SD from two independent experiments (n = 50 cells for each experiment).

(K) Effect of condition medium on cell death of Rpl24-knockdown cells. MDCK-pTR Rpl24-shRNA1 cells were cultured alone with tetracycline under confluent conditions in the presence of condition medium from co-cultured normal and Rpl24-knockdown cells. Cell death was analyzed with SYTOX-dye. Data are mean ±SD from two independent experiments (n = 100 cells for each experiment).

(C, E, and G) Scale bar, 10 μm.

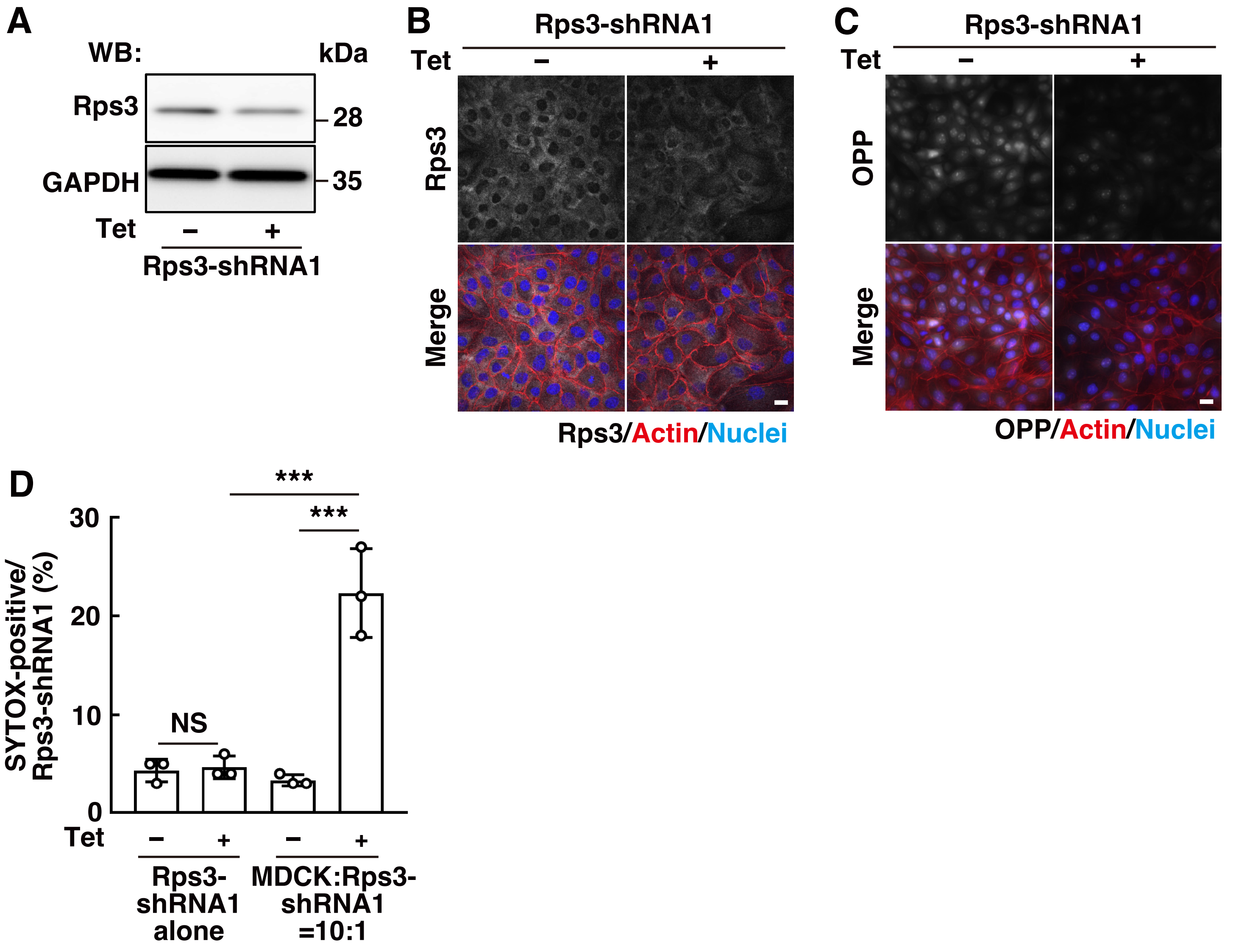

Supplementary Figure 2 Song et al.

Figure S2. Effect of Rps3 knockdown on protein synthesis and cell competition, related to Figure 1

(A) Rps3 knockdown induced by tetracycline addition in MDCK-pTR Rps3-shRNA1 cells. MDCK-pTR Rps3-shRNA1 cells were incubated with or without tetracycline for 72 h, and cell lysates were examined by western blotting using anti-Rps3 and anti-GAPDH antibodies.

(B and C) Effect of Rps3 knockdown on Rps3 immunofluorescence (B) or OPP fluorescence (C). MDCK-pTR Rps3-shRNA1 cells were cultured with or without tetracycline for 72 h under confluent conditions. Cells were then stained with anti-Rps3 antibody or OPP (white) together with Alexa Fluor 568-phalloidin (red) and Hoechst (blue).

(D) Cell death of Rps3-knockdown cells co-cultured with normal cells. MDCK-pTR Rps3-shRNA1 cells were cultured alone or co-cultured with normal MDCK cells with or without tetracycline under confluent conditions. Cell death was analyzed with SYTOX-dye. Data are mean ±SD from three independent experiments (n = 100 cells for each experiment). ***$p < 0.001$, NS, not significant (one-way ANOVA with Tukey's test).

(B and C) Scale bar, 10 μm.

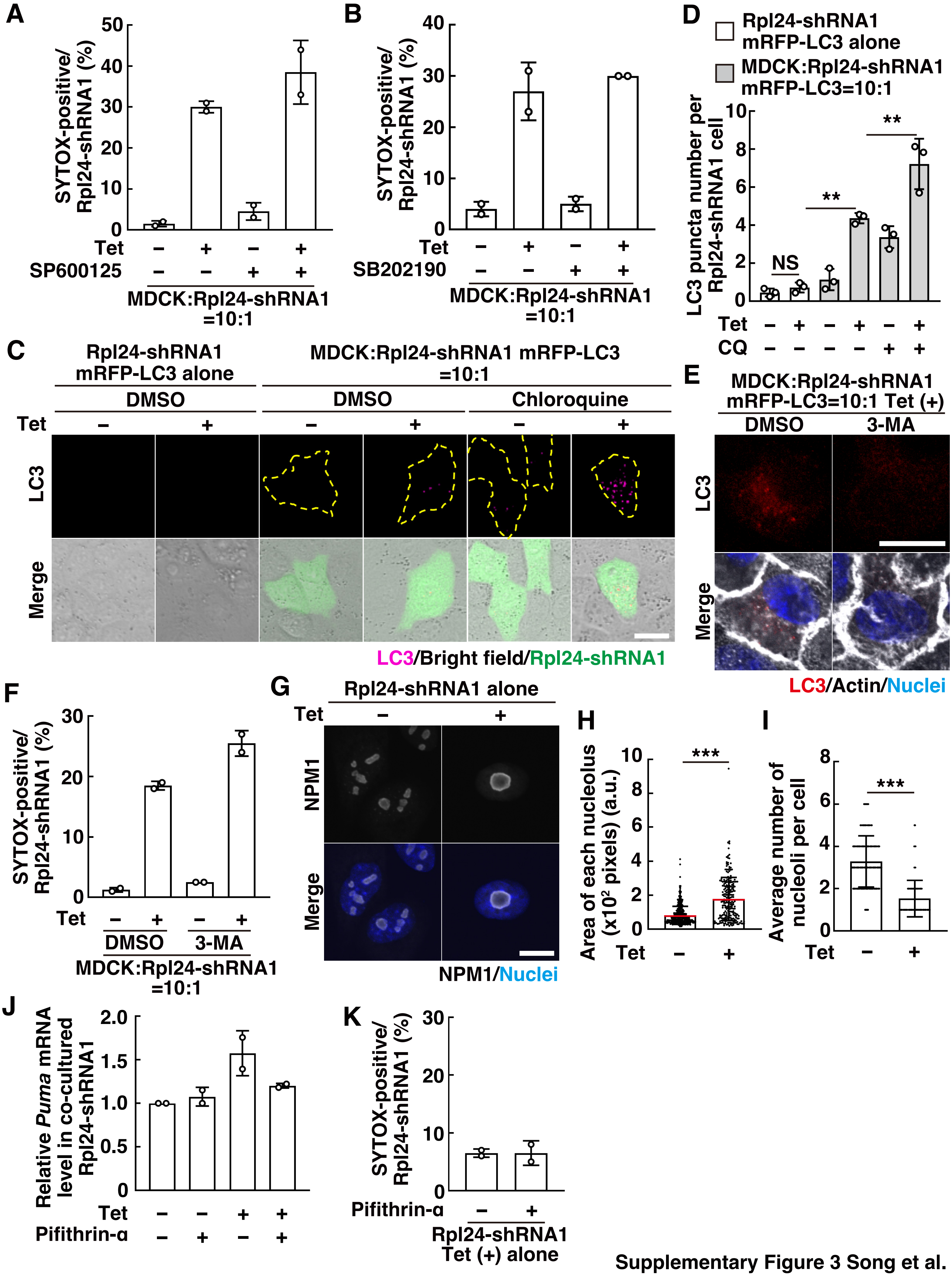

Supplementary Figure 3 Song et al.

Figure S3. Effect of inhibitor for JNK, p38 MAPK, or autophagy on cell competition-mediated death of Rpl24-knockdown cells, related to Figure 2

(A and B) Effect of JNK inhibitor (A) or p38 MAPK inhibitor (B) on cell death of Rpl24-shRNA cells. MDCK-pTR Rpl24-shRNA1 cells were co-cultured with normal MDCK cells with or without tetracycline in the presence of the JNK inhibitor SP600125 (A) or the p38 MAPK inhibitor SB202190 (B). Cell death was analyzed with SYTOX-dye. Data are mean ± SD from two independent experiments (n = 100 cells for each experiment).

(C and D) Effect of Rpl24 knockdown on LC3-positive autophagic puncta. MDCK-pTR Rpl24-shRNA1 mRFP-LC3 cells (GFP-positive) were cultured alone or co-cultured with normal MDCK cells with or without tetracycline and/or chloroquine (CQ) under confluent conditions. (C) The yellow dashed line indicates a Rpl24-shRNA1 cell. Note that, GFP-signals are not shown in 'Rpl24-shRNA1mRFP-LC3 alone' to clearly delineate the contour of each cell. (D) Quantification of the number of LC3-positive puncta. Data are mean ±SD from three independent experiments (n = 50 cells for each experiment). $**p < 0.01$, NS, not significant (one-way ANOVA with Tukey's test).

(E) Effect of the autophagy inhibitor 3-MA on LC3-positive puncta in Rpl24-knockdown cells. MDCK-pTR Rpl24-shRNA1 mRFP-LC3 cells were co-cultured with normal MDCK cells with tetracycline in the presence or absence of 3-MA. Cells were then stained with Alexa Fluor 647-phalloidin (white) and Hoechst (blue).

(F) Effect of 3-MA on cell death of Rpl24-shRNA cells. MDCK-pTR Rpl24-shRNA1 cells were co-cultured with normal MDCK cells with or without tetracycline and/or 3-MA. Cell death was analyzed with SYTOX-dye. Data are mean ±SD from two independent experiments (n = 100 cells for each experiment).

(G–I) Effect of Rpl24 knockdown on the nucleolus. MDCK-pTR Rpl24-shRNA1 cells were cultured alone with or without tetracycline at low density. Cells were then stained with anti-nucleolar marker nucleophosmin (NPM)1 antibody (white) and Hoechst (blue). (H and I) Quantification of the area of each nucleolus (H) and the number of nucleoli per cell (I). Data are mean ±SD from 150 cells from three independent experiments. $***p < 0.001$ (unpaired two-tailed Student's t test).

(J) qPCR analysis of p53-downstream target expression in pifithrin-α-treated Rpl24-shRNA cells. MDCK-pTR Rpl24-shRNA1 cells were co-cultured with normal MDCK cells with or without tetracycline and/or pifithrin-α. Rpl24-shRNA1 cells were then collected by FACS, and the RNAs were examined by qPCR. Data are mean ±SD from two independent experiments.

(K) Effect of pifithrin-α on cell death of monocultured Rpl24-knockdown cells. MDCK-pTR Rpl24-shRNA1 cells were cultured alone with tetracycline in the absence or presence of pifithrin-α under confluent conditions. Cell death was analyzed with SYTOX-dye. Data are mean ±SD from two independent experiments (n = 100 cells for each experiment).

(C, E, and G) Scale bar, 10 μm.

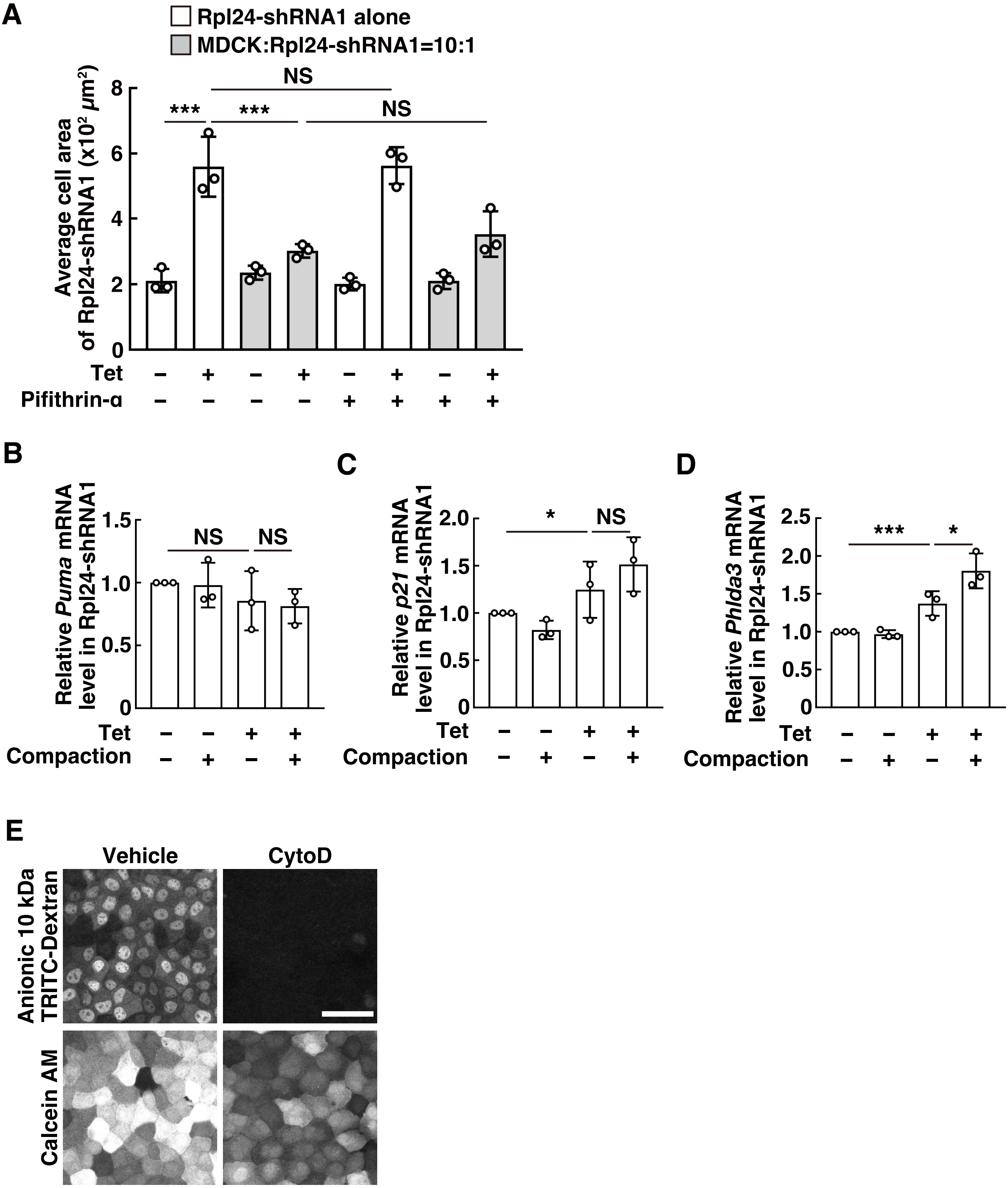

Supplemantary Figure 4 Song et al.

Figure S4. Effect of the p53 inhibitor pifithrin-α on cell area of Rpl24-knockdown cells, related to Figure 3

(A) MDCK-pTR Rpl24-shRNA1 cells were cultured alone or co-cultured with normal MDCK cells with or without tetracycline and/or pifithrin-α under confluent conditions. Data are mean ±SD from three independent experiments (n = 50 cells for each experiment). ***p < 0.001, NS, not significant (one-way ANOVA with Tukey's test).

(B–D) qPCR analysis of p53-downstream target expression in compacted Rpl24-shRNA cells. MDCK-pTR Rpl24-shRNA1 cells were cultured alone with or without tetracycline on the stretchable PDMS membrane. Cell compaction assay was then performed, and the RNAs were examined by qPCR. Data are mean ±SD from three independent experiments. * p < 0.05, ***p < 0.001, NS, not significant (one-way ANOVA with Tukey's test).

(E) Effect of Cytochalasin D (CytoD) treatment on cell-surface tension examined by ELASTomics. Representative fluorescence images of anionic 10 kDa TRITC-dextran after the ELASTomics procedure are shown. MDCK cells were cultured under confluent conditions in the absence or presence of CytoD (4 μM) for 1 h, followed by the ELASTomics procedure to assess cell-surface tension. It should be noted that because the track-etched membrane is semi-transparent and makes transmitted-light visualization difficult, after ELASTomics, cells were counterstained with cell-permeable Calcein AM dye to facilitate cell visualization.

(E) Scale bar, 50 μm.

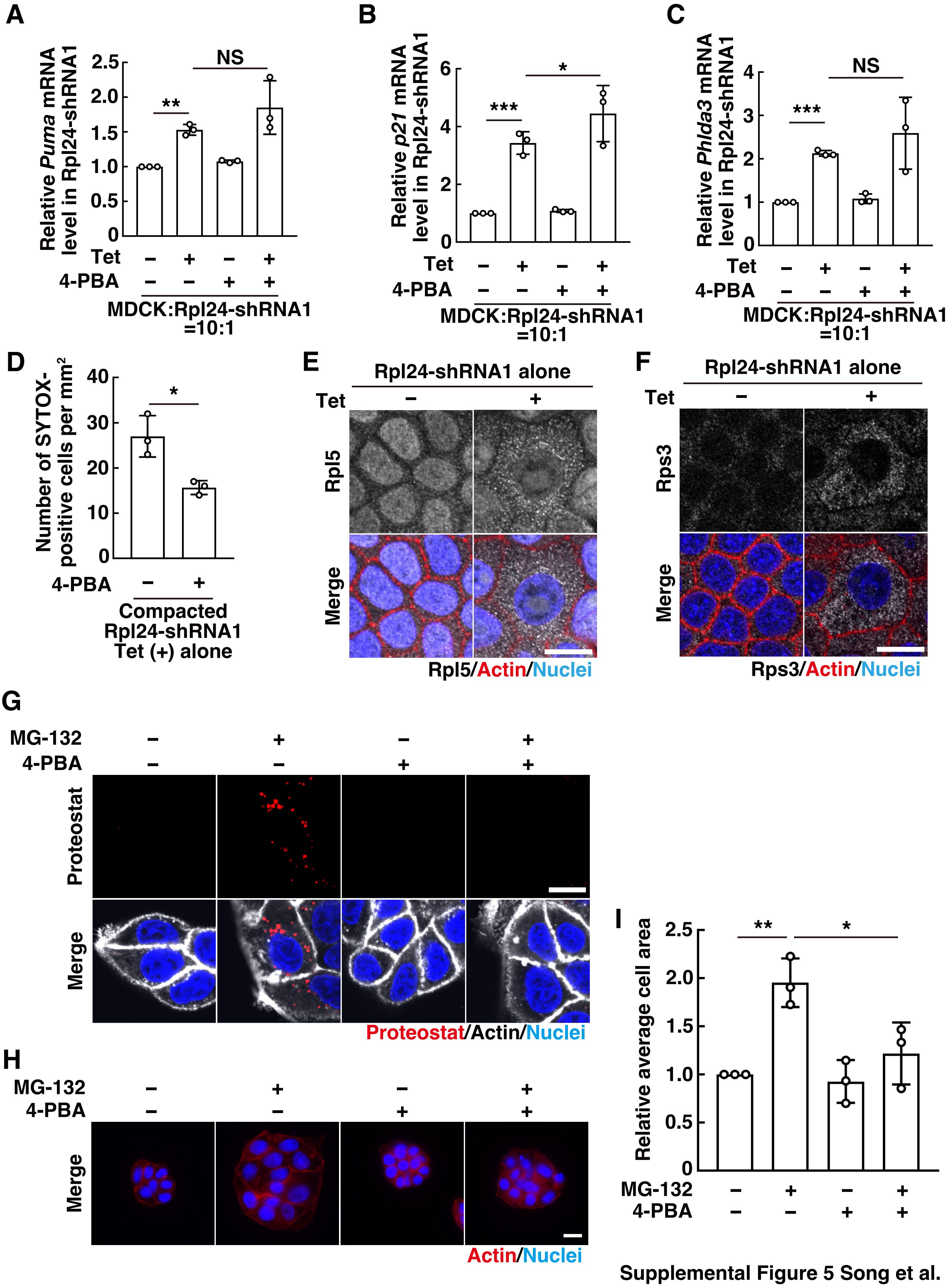

Supplemental Figure 5 Song et al.

Figure S5. Effect of 4-PBA on p53-downstream target expression and compaction-mediated cell death in Rpl24-knockdown cells, related to Figure 4

(A–C) qPCR analysis of p53-downstream target expression in 4-PBA-treated Rpl24-shRNA cells. MDCK-pTR Rpl24-shRNA1 cells were co-cultured with normal MDCK cells with or without tetracycline and/or 4-PBA. Rpl24-shRNA1 cells were then collected by FACS, and the RNAs were examined by qPCR. Data are mean ±SD from three inde pendent experiments. *$p < 0.05$, **$p < 0.01$, ***$p < 0.001$, NS, not significant (one-way ANOVA with Tukey's test).

(D) Effect of 4-PBA on compaction-mediated cell death in Rpl24-knockdown cells. MDCK-pTR Rpl24-shRNA1 cells were cultured alone with tetracycline in the absence or presence of 4-PBA on the stretchable PDMS membrane. Cell compaction assay was then performed, and cell death was analyzed by time-lapse observation using SYTOX-dye. Data are mean ±SD from three independent experiments. *$p < 0.05$ (unpaired two-tailed Student's t test). The number of SYTOX-positive cells were counted within three fields (0.64 x 0.64 mm^2) for each experiment.

(E and F) Effect of Rpl24 knockdown on subcellular localization of Rpl5 (E) and Rps3 (F). MDCK-pTR Rpl24-shRNA1 cells were cultured alone with or without tetracycline under confluent conditions. Cells were then stained with anti-Rpl5 or anti-Rps3 antibody (white), Alexa fluor 568-phalloidin (red), and Hoechst (blue).

(G) Effect of 4-PBA on protein aggregates in MG-132-treated cells. MDCK-pTR Rpl24-shRNA1 cells were cultured alone without tetracycline in the absence or presence of MG-132 and/or 4-PBA at low density and stained with Proteostat (red), Alexa fluor 647-phalloidin (white), and Hoechst (blue).

(H and I) Effect of 4-PBA on cell area of MG-132-treated cells. MDCK-pTR Rpl24-shRNA1 cells were cultured alone without tetracycline in the absence or presence of MG-132 and/or 4-PBA at low density and stained with Alexa fluor 568-phalloidin (red) and Hoechst (blue). (I) Quantification of cell area. Data are mean ±SD from three independent experiments (n = 30 for each experiment). *$p < 0.05$, **$p <0.01$ (one-way ANOVA with Tukey's test).

(E–H) Scale bar, 10 μm.

Oligonucleotides	Sequence
Rpl24-shRNA1 Forward	GATCCCCGGCTAAGCAAGCATCTAAATTCAAGAGATTTAG ATGCTTGCTTAGCCTTTTTC
Rpl24-shRNA1 Reverse	TCGAGAAAAAGGCTAAGCAAGCATCTAAATCTCTTGAATT TAGATGCTTGCTTAGCCGGG
Rpl24-shRNA2 Forward	GATCCCCTGGTGGAAAACGCTAAGTTTTCAAGAGAAACT TAGCGTTTTCCACCATTTTTC
Rpl24-shRNA2 Reverse	TCGAGAAAAATGGTGGAAAACGCTAAGTTTCTCTTGAAA ACTTAGCGTTTTCCACCAGGG
Rps3-shRNA1 Forward	GATCCCCCGGTGCAAATTTCCAAGAATTCAAGAGATTCTT GGAAATTTGCACCGTTTTTC
Rps3-shRNA1 Reverse	TCGAGAAAAACGGTGCAAATTTCCAAGAATCTCTTGAATT CTTGGAAATTTGCACCGGGG
Primer, GAPDH Forward	AACATCATCCCTGCTTCCAC
Primer, GAPDH Reverse	GACCACCTGGTCCTCAGTGT
Primer, Puma Forward	GTGGACATCAGGGACTTGGG
Primer, Puma Reverse	GGGCTGGTAGTCCAGTATGC
Primer, p21 Forward	CATCCCTCATGGCAGCAAGG
Primer, p21 Reverse	TCAAAGTTCCATCGCTCCCG
Primer, Phlda3 Forward	CCACATCTACTTCACGCTGG
Primer, Phlda3 Reverse	CTGCTGGTTCTTGAACTTGACC

Table S1. Oligonucleotides used in this study. Related to Key Resources Table.
